# Forecasting Effusive Dynamics and Decompression Rates by Magmastatic Model at Open-vent Volcanoes

**DOI:** 10.1038/s41598-017-03833-3

**Published:** 2017-06-20

**Authors:** Maurizio Ripepe, Marco Pistolesi, Diego Coppola, Dario Delle Donne, Riccardo Genco, Giorgio Lacanna, Marco Laiolo, Emanuele Marchetti, Giacomo Ulivieri, Sébastien Valade

**Affiliations:** 1 Dipartimento di Scienze della Terra, Università di Firenze, 50121 Firenze, Italy; 2 Dipartimento di Scienze Mineralogiche e Petrologiche, Università di Torino, 10124 Torino, Italy; 3 Dipartimento di Scienze della Terra e del Mare, Università di Palermo, Palermo, Italy

## Abstract

Effusive eruptions at open-conduit volcanoes are interpreted as reactions to a disequilibrium induced by the increase in magma supply. By comparing four of the most recent effusive eruptions at Stromboli volcano (Italy), we show how the volumes of lava discharged during each eruption are linearly correlated to the topographic positions of the effusive vents. This correlation cannot be explained by an excess of pressure within a deep magma chamber and raises questions about the actual contributions of deep magma dynamics. We derive a general model based on the discharge of a shallow reservoir and the magmastatic crustal load above the vent, to explain the linear link. In addition, we show how the drastic transition from effusive to violent explosions can be related to different decompression rates. We suggest that a gravity-driven model can shed light on similar cases of lateral effusive eruptions in other volcanic systems and can provide evidence of the roles of slow decompression rates in triggering violent paroxysmal explosive eruptions, which occasionally punctuate the effusive phases at basaltic volcanoes.

## Introduction

Volcanic eruptions can be considered as the mechanism that the crust uses to restore the equilibrium lost when lithostatic pressure is increased by the arrival of new magma from deeper depths. However, recent measurements of ground deformation and topographic leveling^1–3^ during effusive eruptions suggest that this well-founded concept of physical volcanology may not always be valid. In fact, the classic view of the effusive eruptions driven by the elastic response of a deep magma chamber^4^ could be difficult to apply to open-vent volcanoes, which act as the release valves of deep excess of pressure. In addition, open-vent volcanoes often exhibit a poorly understood rapid transition from explosive to effusive activity, and vice versa, making it very difficult to predict their general behavior and, as a consequence, generating a large degree of uncertainty in the assessment of their related risk.

Although Stromboli (Italy) is a well-known volcano, famous worldwide for its persistent but moderate explosive activity, data collected during the last four effusive eruptions require a new scenario to explain the dynamics of the effusive mechanisms. The continuous explosive activity at Stromboli is based on a delicate equilibrium between a shallow, gas-poor, high-porphyricity (HP), shoshonitic basaltic magma and a deep-seated, gas-rich, low-porphyricity (LP) magma continuously refilling a shallow reservoir^5–8^ at a constant rate of ~0.3 m^3^ s^−1^ (ref. 9). From time to time, periods of higher magma recharge from the deep-seated reservoir can occur, and these are marked by an increased intensity of the Strombolian activity at the summit craters, which can lead to effusive eruptions.

During the last 30 years, four effusive eruptions occurred at Stromboli volcano, in 1985, 2003, 2007 and 2014. All of the effusive eruptions were fed by lateral vents and emplaced large volumes (~10^6^–10^7^ m^3^) of lava in the horseshoe-shaped depression of the Sciara del Fuoco. In all cases, the ordinary Strombolian activity at the summit vents suddenly ceased, returning only after several months, at the end of the effusive phase. Despite these similar features, each eruption was characterized by different durations and different total amounts of erupted lava and, more importantly, each event posed distinctive hazards.

Given the position of the Sciara del Fuoco, one of the most important aspects of the effusive eruptions is the possible occurrence of flank instability. An intense phase of landslide activity along this flank and/or a partial sector collapse of the summit cones into the sea, such as the 25–30 × 10^6^ m^3^ landslide that occurred on 30 December 2002^10^, can generate tsunami waves able to strike the island and the coasts of Sicily and Calabria.

Although basaltic systems typically exhibit slow decompression rates during their effusive phases, two (2003 and 2007) out of four recent eruptions were also marked by the occurrence of a violent paroxysmal explosion^11–14^. The mechanisms by which the basaltic systems erupt explosively have been tentatively explained by eruption products^15–17^, conduit geometry^18^, bubble nucleation^19^, the dynamics of magma degassing^20^ and by analogue studies^21, 22^. However, how slow decompression rates (< 400 Pa s^−1^) of basaltic magmas can cause a rapid shift from an effusive to explosive regime^23–25^ or why contemporaneous effusive-explosive activity can occur^26, 27^ are still matters of debate.

A detailed analysis and comparison of the geophysical observations collected during the most recent effusive episodes (2003, 2007, 2014), along with information available from the literature for the older (1985) event, are used here to derive the general dynamics driving the effusive eruptions. We show that, despite the different durations and eruption volumes, all the phenomena have a common origin, and the four eruptions can be explained with the same effusive dynamics. Finally, we propose a model that can be applied to other lateral effusive eruptions, allowing for the investigation of the dynamics of effusive-to-explosive magma partitioning. The results presented in our work have considerable implications for hazard management.

## Results

**Figure 1:**
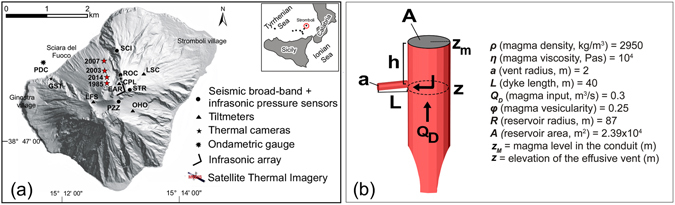
(**a**) Shaded relief map of Stromboli showing the permanent geophysical network deployed by the Laboratorio di Geofisica Sperimentale (University of Florence) since 2003. The red stars indicate the positions of the effusive vents during the four eruptions that occurred in 1985, 2003, 2007 and 2014 along the flank of Sciara del Fuoco. The map of Stromboli was created using Matlab ver. 7.5.0.338 (R2007b). (**b**) A sketch of the shallow reservoir with the main parameters used to define the geometry of the feeding system considered in the modeling.

Most of the data used in this work were collected by the permanent monitoring network deployed by the Laboratorio di Geofisica Sperimentale (LGS) of the University of Florence (UNIFI). The network has been designed to work as an integrated multiparametric geophysical tool, focused on detecting the most shallow (<1 km) and rapid (<1 s) magma dynamics. It was deployed during the 2002–03 eruption, and has been continuously expanded ever since^1, 28^. The network consists of four seismo-acoustic stations located in the upper part of the volcano, equipped with three-component broad-band seismometers and one infrasonic pressure sensor, both sampled at 100 Hz. A five-component L-shaped infrasonic array, two thermal infrared cameras (FLIR A20) and three borehole tiltmeters sampled at 1 Hz were used. One tide gauge was installed in front of the Sciara del Fuoco to monitor the occurrence of possible submarine landslides and tsunami waves (Fig. 1a). In addition, thermal images from satellites using the Moderate Resolution Imaging Spectroradiometer (MODIS) sensor onboard the Terra and Aqua satellites were analyzed automatically in real-time in collaboration with the University of Torino^29^.

### Effusion Rates and Total Volumes

**Figure 2:**
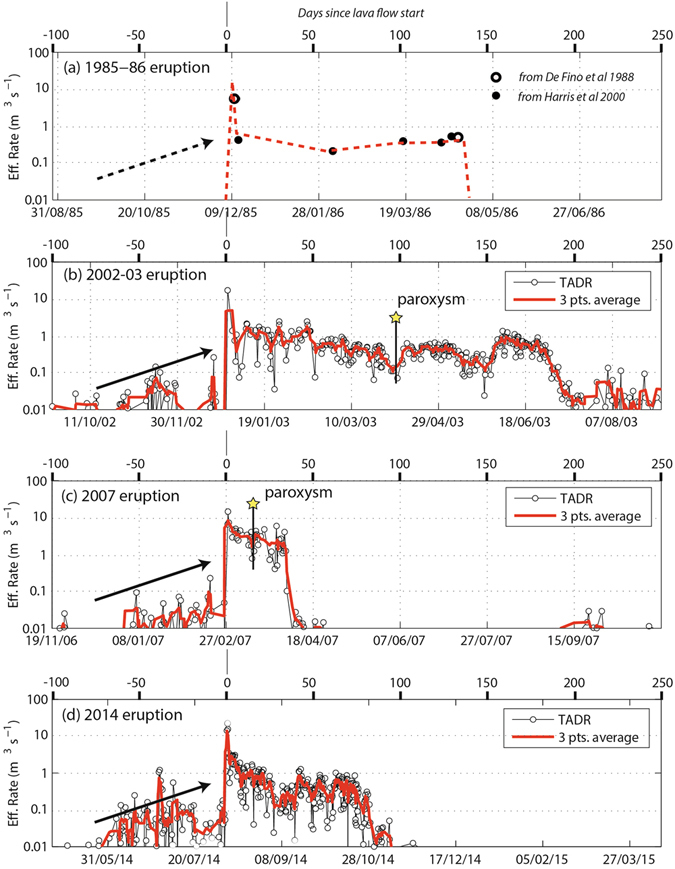
Effusive trends relative to the (**a**) 1985, (**b**) 2003, (**c**) 2007 and (**d**) 2014 eruptions of Stromboli. The datasets are represented on the logarithmic scale and were derived from the literature^30, 38^ (**a**) and MODIS data^25, 29, 31, 32, 34^ (**b**–**d**). The time-series span from day −100 to day +250, with day 0 representing the beginning of each eruption. The red lines outline the main trends, all of which are characterized by a sharp increase at the eruption onset, followed by a general decline of effusion rates toward the end the eruptions. The yellow stars mark the occurrence of the two paroxysms that occurred on 5 April, 2003, and 15 March, 2007, respectively. Note the general increase of the output rates recorded during the weeks preceding the onset of each eruption (black arrows).

The last four effusive eruptions, occurring in 1985^30^, 2003^25^, 2007^28, 31^ and 2014^32^, are all marked by the end of the explosive activity visible in the summit craters and by the opening of an effusive lateral vent at the base of the NE cone (Fig. 1), within Sciara del Fuoco, at elevations ranging between 400 and 680 m above sea level (a.s.l.). The lateral effusive vents are the result of the propagation of a lateral dyke from the main central conduit towards the Sciara del Fuoco (Fig. 1). The four eruptions lasted from 35 to 205 days and produced volumes of lava^1, 30–35^ ranging between ~6 × 10^6^ and ~11 × 10^6^ m^3^, corresponding to ~4 × 10^6^ and ~7 × 10^6^ m^3^ of dense rock equivalent (DRE) magma when a vesicularity of ~30 vol.% is considered (Fig. 2, Table 1).

**Table 1:** Parameters related to the eruptive crises.

	1985	2003	2007	2014
Elevation of the effusive vent (m a.s.l.)	680 m	580 m	400 m	650 m
H reservoir (m)	70	170	350	100
Duration (days)	141	205	35	107
Mean Effusion Rate (m^3^ s^−1^)	0.45	0.61	3.16	0.60
Erupted Volume (Mm^3^)	5.5	10.8	9.5	5.5
Effusion Rate days 0 to 35 (m^3^ s^−1^)	0.98	1.59	3.16	1.26
Volume days 0 to 35 (Mm^3^)	2.9	4.8	9.5	3.8
Effective reservoir radius (m, from total volume)	87	87	87	87
Density (kg m^−3^)	2950	2950	2950	2950
Viscosity (Pa s)	10^4^	10^4^	10^4^	10^4^
Effective vent radius (m)	2	2	2	2
Dyke length (m)	40	40	40	40
Decompression Rate (Pa s^−1^) (ϕ = 0.25)	5.9 ± 0.6	14.3 ± 1.6	29.3 ± 3.5	8.4 ± 0.9

Parameters of the effusive eruptions and data used in the gravity-driven discharge model (see Figure 1b). As reported in the text, most of the values (e.g., elevation of the effusive vents, vent radius, durations of the effusive eruptions) were directly observed during the effusive eruptions, while others (e.g., the total lava volumes, effusion rates) are represented as ranges because of both their uncertainties and natural variabilities; their associated errors are reported in the text.

**Figure 3:**
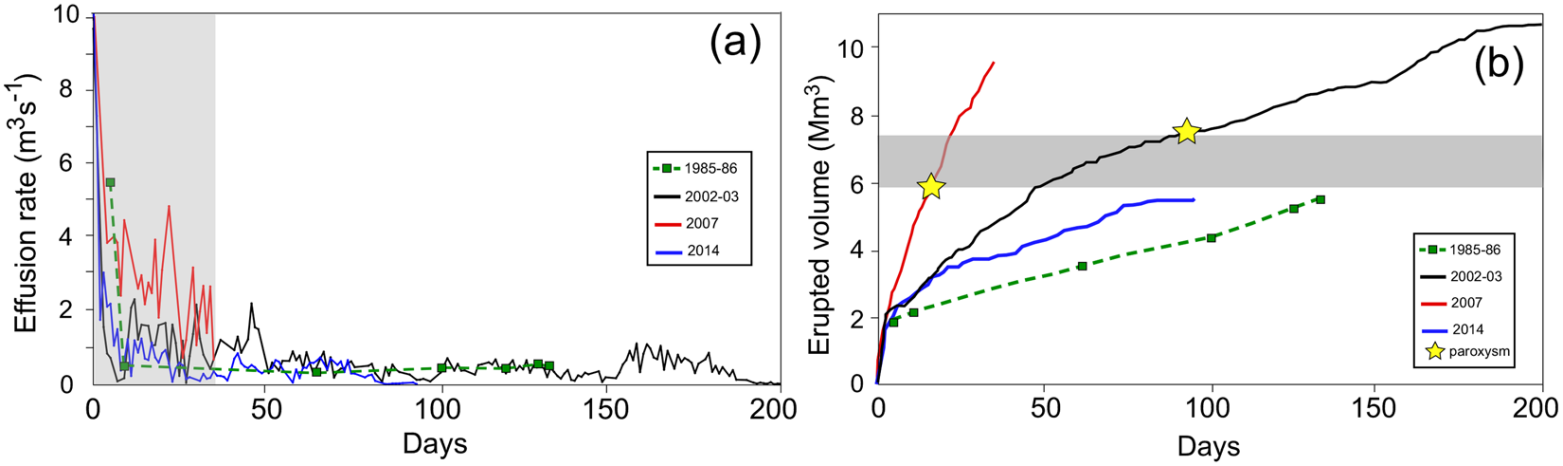
(**a**) Comparison between the effusion rate trends relative to the 1985 (dashed green), 2003 (black), 2007 (red) and 2014 (blue) eruptions. The shaded gray area represents the exponentially decreasing trend of the first 35 days. (**b**) Cumulative volumes for the same eruptions. The gray line represents the threshold of 6.5 ± 1 × 10^6^ m^3^ of erupted lava, after which the paroxysmal explosions (yellow stars) occurred in 2003 and 2007, at 15 and 90 days from the eruption onset, respectively.

Thermal satellite images from MODIS show that the last three recent eruptions (2003, 2007, 2014) started with high value effusion rates (Fig. 2), with daily mean values of 14–22 m^3^ s^−1^. The effusion rates have been calculated from the radiative power (in Watt) of the MODIS images using the radiant density approach, which depends on the lava silica content^36^. This approach allows for the estimation of near-real-time effusion rates, with a mean error of about the 30%^29, 36^. However, at Stromboli, the ground-based independent measurements of lava volumes erupted during the 2003 and 2007 eruptions^31, 33, 37^ allow for better constraints of the satellite thermal images, reducing the error down to ~10%, which is mainly related to the quality of the satellite images (e.g., cloud cover, zenith angle). The effusion rates rapidly decay, following an almost exponential trend^1, 25, 31–34^ until they reach a stable value in the range 0.4–0.8 m^3^ s^−1^ after <5 days from the onset (Fig. 3).

Data available in the literature indicates that the 1985 eruption was characterized by an initial effusion rate probably of ~5–6 m^3^ s^−1^, followed by a rapid decrease to 0.4 m^3^ s^−1^ during the first week^30, 38^. Despite this general common trend, the mean effusion rate $\overline{Q}$ derived by thermal imagery:

1 $$\overline{Q}=\frac{1}{T}{\int }_{t=0}^{T}Q(t)dt$$

and the total volume *V* of magma erupted:

2 $$V={\int }_{0}^{T}Q(t)dt$$

both of which calculated over the whole duration (*T*) of each effusive eruption, are significantly different. While the 1985, 2003 and 2014 effusive eruptions are characterized by low mean effusion rates of 0.45, 0.61 and 0.60 m^3^ s^−1^, respectively, the 2007 eruption shows a higher mean effusion rate of 3.16 m^3^ s^−1^ (Fig. 3, Table 1).

The gradual decrease of the effusion rate is always accompanied by an increase in the number of explosions recorded daily at the summit craters^31^, reflecting the closure of the effusive vent in response to the progressive reduction of the pressure within the lateral feeding fracture (or dyke) and the gradual magma ascent along the central conduit^25, 39^.

### Ground deformation at the effusive onset

**Figure 4:**
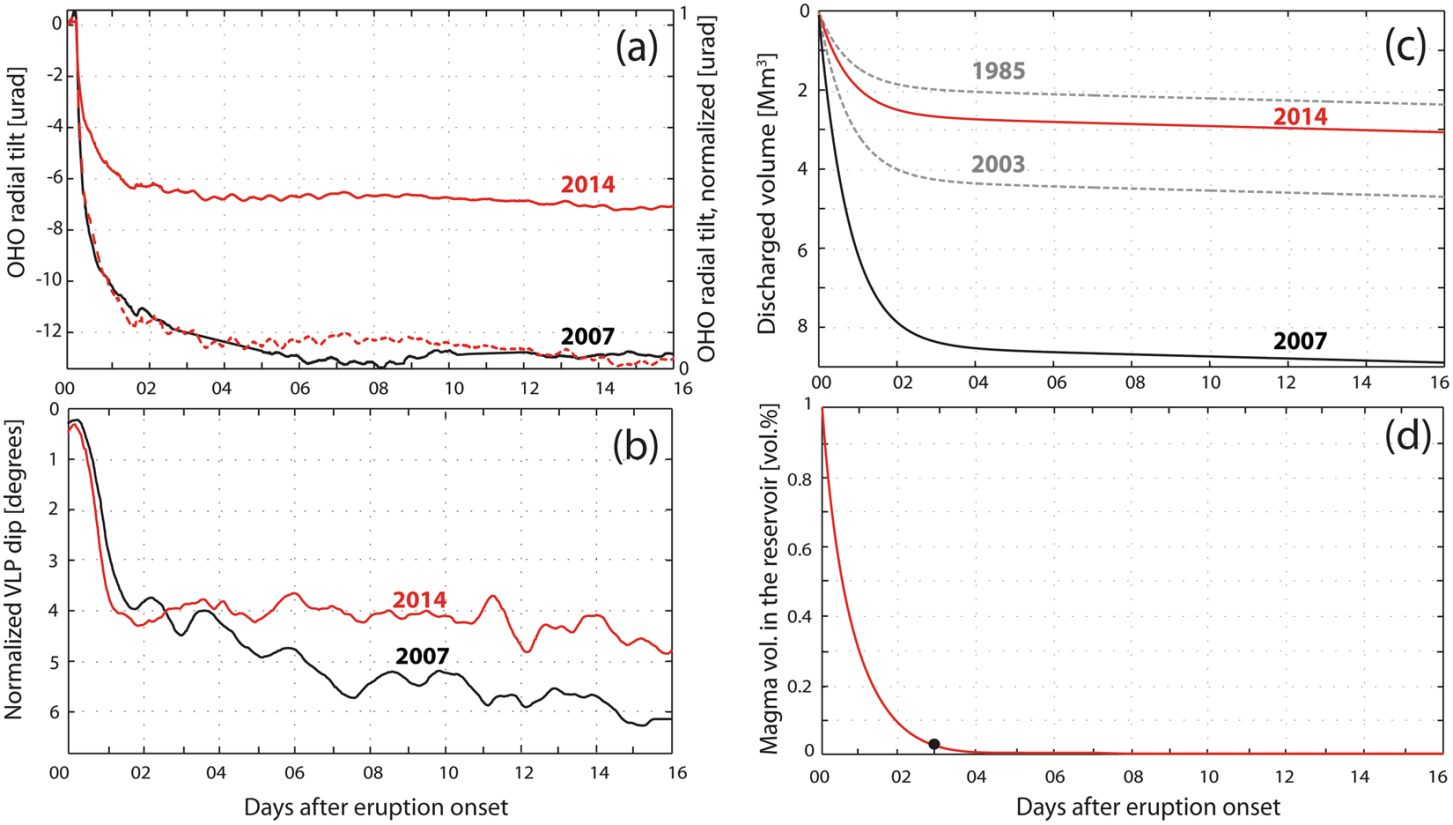
(**a**) Ground deformation during the 2007 (black) and 2014 (red) effusive eruptions, recorded by a borehole tiltmeter at the station OHO. The normalized ground deformation (dashed red for the 2014) is also shown. Tiltmeter locations are reported in Fig. 1. (**b**) The normalized VLP dip for the 2007 and 2014 eruptions. (**c**) Discharged volumes during the 2014 (red) and 2007 (black) eruptions. 1985 and 2003 trends are also shown (dashed gray) for comparison. (**d**) Percentage of the magma volume drained from the reservoir, modeled using equation (5). Despite the volume of magma considered, the model predicts that the drainage of the volume stored in the reservoir always follows the same exponential discharge trend. The largest decompression rate coincides with the inflection point (black dot) of the discharge trends, which indicates that 98% of the magma volume is drained out of the reservoir in the first 3 days of the effusive eruption (equation (10)).

The 2007 and 2014 effusive eruptions were also monitored by a network of borehole tiltmeters deployed at distances close (<800 m) to the summit craters. Small ground inflations of ~1 µrad were detected by tiltmeters only few hours before the effusive onsets, and are associated with an increase in the landslide activity along the Sciara del Fuoco^32, 40, 41^. As soon as the lava started to flow out of the lateral effusive vent, the tiltmeters detected a clear deflation of the volcanic edifice which, over a period of 48 hours, reached 7 µrad during the 2014 lava effusion^32^, and was double that, at 13 µrad, during the 2007 eruption^40^. Despite the different amplitudes and different durations of the eruptions, the ground deformation, once normalized, shows exactly the same deflation trend, implying a similar magma discharge mechanism (Fig. 4a).

### Magma Drainage Dynamics

The rapid deflation of the ground at the effusion onset coincides, both in the 2007 and 2014 eruptions, with the deepening of the Very-Long-Period (VLP) seismic source (Fig. 4b). No tilt observations are available for the 2003 eruptions, but the position of the seismic VLP source, measured ~1 month after the eruption onset, was ~5° deeper than that before the eruption^42^, suggesting a similar behavior. In addition, the trends of the ground deformations in the 2007 and 2014 events followed that of the effusion rate^1, 32^, indicating that the deflation of the volcanic edifice is somehow responding to the magma drainage of the reservoir.

Interestingly, the ground deformation and effusion rates are both linked to the deepening of the seismic VLP source, which seems consistent with a lowering of the magma level in the central conduit^1, 25, 32^. The drop of the magma level during the effusive phase can, in fact, indicate either that the effusive vent and the shallow conduit were decoupled from the deep magma reservoir or that the magma supply rate at depth during the effusive eruption was not large enough to sustain the magma column in the central conduit^1, 32^. In this second case, observations point to a model of effusive eruptions controlled by the decrease of magmastatic pressure due to the emptying of the shallow reservoir.

### Effusion Rate and Position of the Effusive Vents

**Figure 5:**
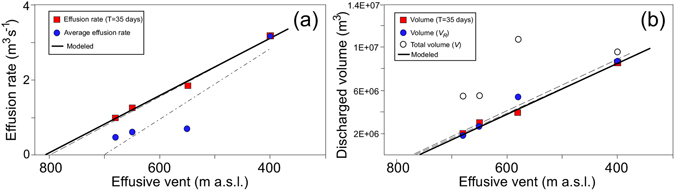
(**a**) Correlation between the elevations of the effusive vents and effusion rates (averaged over the whole duration and over the same 35 days’ time) during the studied effusive eruptions. (**b**) The discharge volume *V*_*R*_ versus the elevation of the effusive vent is calculated, considering *Q*_*D*_ = 0.3 m^3^ s^−1^. Note that the intercepts of the trend lines and the lower axis coincide with the elevations (~750–780 m a.s.l.) of the active craters. Open circles refer to the total erupted volumes, *V*, assuming *Q*_*D*_ = 0 m^3^ s^−1^ in equation (5). The modeled trends are also reported for comparison as the solid bold lines.

Despite the different durations of the four effusive episodes, the hypothesis of a shallow origin of the erupted magma volume finds strong support in the good inverse correlation (R^2^ = 0.91) between the mean effusion rates ($\overline{Q}$) and the elevation (*z*) of the effusive vents during the four eruptions (Fig. 5a). The correlation increases to R^2^ = 0.99 (Fig. 5a) when the volume erupted in the same time interval, e.g., *T* = 35 days (equivalent to the shortest duration of the four effusive eruptions), is considered (Table 1). In this case, the best linear fit, $\overline{Q}=-0.0077z+6.18$, predicts a mean effusion rate of ~6.2 m^3^ s^−1^, if the effusive vent is located at sea level (*z* = 0 m), and it gives a maximum possible elevation *z* = 803 m a.s.l of the effusive vent for $\overline{Q}$ = 0 m^3^ s^−1^. However, Strombolian activity is constantly fed by a small magma supply rate of 0.3 m^3^ s^−1^ (ref. 9), which moves the maximum position of the effusive vent to 764 m a.s.l. This fits the position of the summit crater terrace remarkably well (750–780 m).

### Modeling the Discharge Rate

The link between effusion rate ($\overline{Q}$) and elevation of the effusive vent (*z*) seems difficult to explain via the decompression of a deep reservoir^4^, and instead supports the idea of the shallow effusive dynamics being controlled by the magma column (*h*) stored in the central conduit system above the lateral vent^1^. In this model, the total volume *V* = *V*_*R*_ + *V*_*D*_ of the magma erupted in a given time interval, *T*, is equivalent to the volume *V*_*R*_ drained out of the shallow conduits, with the contributions ${V}_{D}={\overline{Q}}_{D}T$ of the magma constantly supplied from depth at the mean rate ${\overline{Q}}_{D}$ = 0.3 m^3^ s^−1^ (refs 1, 9, 43). Hence, we consider that the drainage of the magma volume (*V*_*R*_) out the shallow reservoir at time *t*, located above the effusive vent, is driven by magmastatic pressure $P(t)=\rho gh(t)(1-\varphi )$ of the central conduit in the lateral effusive dyke, where *ρ* is the magma density and *ϕ* the magma vesicularity (Fig. 1b). The conduit geometry of volcanoes is much more complex than our simple central conduit evolving in a lateral dyke system, which, considering the elasticity of the conduit walls, would probably have significant effects on magma flow dynamics^44^. However, we keep the model as simple as possible, and we consider that the effusion rate *Q*(*t*) is controlled by the Poiseuille flow of the magma, with a viscosity *η* along the lateral rigid dyke of length *L* and with an equivalent effective radius *a*:

3 $$Q(t)=\frac{\pi {a}^{4}\rho g}{8\eta L}[{z}_{m}(t)-z](1-\varphi )$$

In this case, the effusion rate is a function of the elevation difference $h(t)=\lfloor {z}_{m}(t)-z\rfloor$ between the effusive vent (*z*) and the level of the magma (*z*_*m*_) within the shallow central reservoir. Assuming that the volume of the magma ${V}_{R}=A\,[{z}_{m}(0)-z]$ in the reservoir of area *A*, is constantly supplied from depth with a rate ${\overline{Q}}_{D}$:

4 $$-\frac{d{V}_{R}(t)}{dt}=Q(t)-{\overline{Q}}_{D}$$

equation (3) can be written as:

5 $$Q(t)={Q}_{o}\,\exp \,[-\frac{\pi {a}^{4}\rho g(1-\varphi )}{8\eta LA}t]+{\overline{Q}}_{D}$$

where ${Q}_{o}=\pi {a}^{4}\rho g(1-\varphi ){V}_{R}/8\eta LA$ is the initial contribution at *t* = 0 of the magma drained from the shallow reservoir to the total effusion rate. This equation demonstrates how the discharge rate in a gravity-driven effusive eruption follows an exponential trend^1, 3, 32, 34^ (Fig. 4c) and, by solving the integral of *Q*(*t*) ﻿in equation (1), allows for the calculation of the mean rate $\overline{Q}$ of the effusive eruption driven by gravity:

6 $$(\overline{Q}-{\overline{Q}}_{D})T=A[{z}_{m}(0)-z]\cdot \{1-\exp \,[-\frac{\pi {a}^{4}\rho g(1-\varphi )T}{8\eta LA}]\}$$

which, from equation (4), represents in this form the volume of the magma drained out of the shallow reservoir.

The exponent will tend to zero when $\pi {a}^{4}\rho g(1-\varphi )T > 8\eta LA$, and thus the following is true for any duration *T* (in seconds) of the effusive eruption:

7 $$\{1-\exp \,[-\frac{\pi {a}^{4}\rho g(1-\varphi )T}{8\eta LA}]\}=1$$

Therefore, equation (6) can be reduced to:

8 $$(\overline{Q}-{\overline{Q}}_{D})T=A[{z}_{m}(0)-z]$$

which predicts a linear relationship between the volume of magma drained out of the reservoir (*V*_*R*_) and the elevation of the vent (*z*) above sea level if the initial level of the magma, *z*_*m*_(0), is a constant parameter for all of the effusive eruptions.

This model is strongly supported by the good linear fit R^2^ = 0.96 (Fig. 5b) between the volume of the magma drained out of the reservoir (*V*_*R*_ = −23967*z* + 1.852 × 10^7^) and the elevation of the effusive vent (*z*), given a constant deep supply rate of 0.3 m^3^ s^−1^. In addition, from equation (8) we can calculate that the mean area of the shallow magma reservoir is *A* = 2.39 × 10^4^ m^2^, which corresponds to an equivalent effective radius of 87 m.

Considering a density *ρ* = 2950 kg m^−3^, a viscosity *η* = 10^4^ Pa s, a vesicularity *ϕ* = 0.25 and a mean effective radius of the lateral effusive vent *a* = 2 m (refs 1 and 32), we model the discharge rate of the shallow reservoir driven by gravity considering different elevations *z* of the effusive vent. As predicted by equation (6), and in agreement with the observations, the mean effusion rate and the total discharged volume show a perfect linear fit (R^2^ = 0.96) with the topographic position of the effusive vent (Fig. 5), if we assume that the length of the dike/sill remained almost constant with *L* = 40 m.

Interestingly, the linear fit becomes worse (R^2^ = 0.46) when no magma supply from depth (${\overline{Q}}_{D}$ = 0) is considered (open dots in Fig. 5b), but the correlation increases, up to R^2^ = 0.99, when the volume of the magma erupted in the same time interval, e.g., *T* = 35 days, is considered (red squares in Fig. 5b).

### Decompression Rate

The gravity-driven eruption model provides an effusive dynamic that well explains most of the physical observations during the last four eruptions at Stromboli. However, two out of the four eruptions (in 2003 and 2007) have also been characterized by violent “paroxysmal” explosions, resulting in a serious threat to the inhabitants of the island and thus representing an additional major hazard^13^.

These energetic explosive events are believed to be produced by the rapid ascent of parcels of deep (7–10 km) gas-rich primitive LP magma^8, 11, 45, 46^. The connection between these rare explosive paroxysms and the effusive activity suggests that the rise of the gas-rich magma batch from depth could somehow be triggered by the drainage of large volumes of lava from the shallow reservoir^35^. At the times of the paroxysms, on 5 April 2003 and 15 March 2007, the amount of effused lava (Fig. 3b, Table 1) was almost the same, at approximately 6.5 ± 1 × 10^6^ m^3^ (the 4.4 × 10^6^ m^3^ value in (ref. 35) is the DRE value derived, considering a mean vesicularity range of 16–32 vol.%), and this value has thus been suggested to be the threshold amount of magma volume necessary to trigger the paroxysms^35^. This volume was not reached during the 1985 and 2014 eruptions, which emplaced ~82% of the volume necessary to trigger the paroxysm (Fig. 3b, Table 1). However, the difference between the volume threshold and the total amounts of lava in the 1985 and 2014 seem too small to justify the absence of explosive paroxysms during those two eruptions.

Despite the different volumes of lava drained during the 2007 and 2014 eruptions, the ground deformations show similar deflation trends (Fig. 4a), indicating that different decompression rates could have affected the equilibrium of a deep magma chamber. The decompression rate *δP* (Pa s^−1^) of the gravity-driven system can then be expressed in terms of the change in the magmastatic pressure:

9 $$\delta P=\rho g\frac{[{z}_{m}(0)-z]}{\tau }(1-{\rm{\Phi }})$$

where *τ* is the time necessary to empty the shallow reservoir derived from equation (5):

10 $$\tau =-\,\mathrm{log}\,(1-{\rm{\Delta }}{V}_{R})\lfloor \frac{8\eta LA}{\pi {a}^{4}\rho g(1-\varphi )}\rfloor $$

assuming that a critical percentage of the magma volume, ${\rm{\Delta }}{V}_{R}={V}_{R}(t)/{V}_{R}$, is drained out the reservoir. Equation (10) shows that the discharge time is independent from the elevation of the effusive vent, and it is the same for the four eruptions ﻿(Figure 4d)﻿. The largest decompression rate is thus reached at the inflection point *τ* = 3 days of the discharge function (Fig. 4d), which coincides with both the ground deflation measured by the tilt and the drastic lowering of the VLP seismic source (Fig. 4a,b). Equation (10) also reveals that almost 98% of the volume stored in the shallow reservoir is drained in the first 3 days following the eruption onset (Fig. 4d).

Thus, we obtain decompression rates of 5.9 ± 0.6 and 8.4 ± 0.9 Pa s^−1^ for the 1985 and 2014 events, respectively, which are ~>50% lower than decompression rates of 14.3 ± 1.6 and 29.3 ± 3.5 Pa s^−1^ calculated for the 2003 and 2007 eruptions, respectively. These decompression rates are low, but compatible with the slow unloading rates (10^−3^ m s^−1^) calculated to explain the transition from effusive to explosive eruptions^21^.

We suggest that the magmastatic effusive discharge induces a slow decompression rate, capable of perturbing the equilibrium between the shallow and the deep reservoirs. Therefore, we propose that the 1985 and 2014 eruptions, although close to the critical threshold of erupted lava (~6.5 × 10^6^ m^3^), did not trigger the explosive ascent of LP gas-rich magma due to their smaller decompression rates, compared to those of the 2003 and 2007 eruptions.

In addition, the higher rate of decompression in the 2007 (29.3 Pa s^−1^) triggered the explosive paroxysms only 15 days after the effusive onset, whereas, in the 2003 event, the decompression rate was slower (14.3 Pa s^−1^) and the time lag was longer (90 days). The decompression rate during the first 3 days of the eruption could thus induce a delayed exsolution process of the deep portion of the magma column, which could control the timing of the explosion after the eruption onset.

Although the geometry of the dyke length (*L*) and the effective radius of the lateral effusive vent (*a*) can play an important role in this process, our model suggests that the decompression rate during the effusive eruptions at Stromboli is mostly controlled by the elevation of the effusive vents, which varied considerably between the studied events.

## Discussion

Effusive eruptions can be considered to be the response of a magmatic system to recover its equilibrium during higher magma recharge rates from a deep reservoir. These are inherently difficult to predict for open-vent systems, owing partly to their infrequency and to the difficulties of observing important pre-eruptive ground deformations.

The most recent four effusive crises that occurred at Stromboli volcano in the last 30 years show that effusive eruptions are preceded by periods of high explosive Strombolian activity. This was sometimes associated with lava overflows confined within the crater area^32^ and reflect the high level of magma in the conduits, which are the result of the deep magma supply rate. Once started, lateral lava effusion is always accompanied by a rapid (several µrad per day) ground deflation, closely following the effusion rates and remarkably correlated with the lowering of the VLP seismic source (Fig. 4).

Similarly, variations of the lava lake level at Kilauea (Hawaii) were also related to both the ground tilt and the effusion rate variations at the Pu’u’O’o vent, and are explained as being driven by the magmastatic load of this lava lake^2^. More recently, a large lateral eruption of Bardarbunga volcano (Iceland) has shown how the 65 m collapse of the ~3 km large caldera was unexpectedly linked to the effusion rate measured at the Holuhraun vent, which is more than 45 km away^3, 47^. However, neither case provides conclusive evidence indicating whether the contraction of the deep magma chamber or the piston effect of the collapsing caldera/lava lake in the central crater were responsible for the observed effusion rate trends.

Here, we demonstrate that all of the effusive eruptions at Stromboli fit gravity-driven discharge dynamics, controlled by the magmastatic load of the shallow reservoir. Remarkably, effusion rates and eruption volumes show a linear correlation (R^2^ = 0.99) with the above-sea-level elevation of the effusive vents. This correlation is difficult to explain in terms of a contraction of a magma chamber or of elastic discharge dynamics of a deep reservoir^4^. Thus, our result seems to provide strong support for the magmastatic control of the effusive dynamics, presenting a new perspective for our understanding of the lateral effusive eruptions.

The model has an immediate impact on the mitigation and assessment of the hazard of these events, as it allows for the forecasting of the volume of lava that will be erupted, using the relative elevation between the effusive vent and the crater terrace. In addition, the linear correlation suggests that, to trigger effusive eruptions at Stromboli, a necessary condition is that the magma level in the reservoir has to reach an elevation of ~765 m a.s.l., which coincides with the elevation of the crater terrace in the pre-eruptive topographic surface. This explains the increase in explosive activity before all four eruptions (Fig. 2) and indicates that the effusive phases at Stromboli should be always preceded by a period of high magma level in the conduit and thus of high explosivity.

Moreover, the model suggests that the shift between the effusive regime and violent explosive eruptions could be explained in terms of the slow decompression rates. Magma analogue experiments indicate that gas-rich, crystal-poor basaltic magma can experience a delayed bubble nucleation process, leading to considerable super-saturation, and thus triggering magma fragmentation under slow decompression rates^22^. We suggest that, at Stromboli, decompression rates >10 Pa s^−1^ can potentially cause the ascent of the parcels of gas-rich, crystal-poor LP magma responsible for the paroxysmal explosions that occurred during the effusive eruptions in 2003 and 2007.

In this view, the Stromboli volcano, known worldwide for its persistent mild Strombolian activity, provides a new perspective for the comprehension of effusive dynamics and of the role of slow decompression rates with respect to deep magmatic systems.
